# Lifetime intimate partner violence exposure, attitudes and comfort among Canadian health professions students

**DOI:** 10.1186/1756-0500-2-191

**Published:** 2009-09-23

**Authors:** Megan R Gerber, André KW Tan

**Affiliations:** 1 Women's Health, VA Boston Healthcare System, Boston, MA, USA; 2 Harvard Medical School, Boston, MA, USA; 3 Department of Otolaryngology, Queen's University, Kingston, Ontario, Canada

## Abstract

**Background:**

Intimate partner violence (IPV) is a widespread public health problem and training of health professions students has become common. Understanding students' prior knowledge, attitudes and personal exposure to IPV will aid educators in designing more effective curriculum. As interprofessional educational efforts proliferate, understanding differences across disciplines will be critical.

**Findings:**

Students in the schools of Medicine, Nursing and Rehabilitation at a university in Ontario attend an annual daylong interprofessional IPV training. To measure perceived role and comfort with IPV and prior personal exposure, we administered a brief Likert scale survey to a convenience sample of students over three years. 552 students completed the survey; the overall response rate was 73%. The majority (82%) agreed that it was their role to intervene in cases of IPV; however Rehabilitation students expressed lower overall comfort levels than did their peers in other schools (p < .0001). Gender, age and prior training on the subject were not significant predictors of comfort. Seven percent reported lifetime IPV and one-fifth had witnessed IPV, but these exposures did not predict comfort in adjusted logistic regression models.

**Conclusion:**

While the majority of professional students believe it is their role to address IPV in clinical practice, comfort level varied significantly by field of study. More than one fifth of the students reported some personal exposure to IPV. However this did not impact their level of comfort in addressing this issue. Educators need to take students' preexisting attitudes and personal exposure into account when planning curriculum initiatives in this area.

## Background

Intimate partner violence (IPV) is a pattern of coercive behavior in which one person attempts to control another through threats or actual use of physical violence, sexual assault and verbal or psychological abuse. [1] Nearly one-third of Canadian women experience IPV in their lifetime and 21.2% report IPV in the preceding 5 years. [2,3] In Canadian family practice settings, the estimated prevalence is 14.6%. [4] IPV has well-established adverse health effects, [5-7] and results in frequent and regular contact between victims and healthcare providers. [4,8] It has thus become widely accepted that training of healthcare professionals is imperative. [9,10] Yet, the sensitive nature of IPV creates challenges for educators who train health professions students. [11-14] The clear limitations of the medical model to provide a straightforward remedy, or "fix", for this problem may be frustrating to many learners. [13] Given the well-documented difficulty many healthcare providers have with inquiry for IPV, [15-17] it would not be surprising to find a dearth of effective role models available during clinical training.

Further adding to these challenges is the possibility of personal exposure to IPV among students. Students who have been victims may experience a range of responses to IPV curricular content including anxiety, vicarious retraumatization and feelings of helplessness. [11,14] Medical students with personal histories of violence express concern about their future efficacy in aiding patients who have had similar experiences. [18] Nonetheless, students who report histories of abuse favor IPV training. [19] In order to provide effective learner-centered curricula, educators need to understand the potential extent of IPV exposure among students.

Curricula to address IPV have proliferated over the last 15 years [9,20] and are most commonly reported in medical and nursing school settings. [21-24] Fewer citations are found for the field of physical therapy and rehabilitation. [25,26]

The prevalence of IPV among US medical students is between 6-12% for women [18,19] and 7% for men[27,28] In a US study of nursing students, 8% reported experiencing IPV[29] Among practicing physicians and nurses in Ontario, nearly 50% reported either personally experiencing or witnessing a close friend or relative experience abuse. [16] Thus, it is also probable that some proportion of Canadian students will have been exposed to IPV, [11,14,18] but to our knowledge, rates have not been reported in the literature.

### Aims of the Study

The main objectives of the study are to explore how student comfort in addressing IPV is impacted by 1) gender, 2) program of study and 3) prior personal experience or training. A secondary aim was to measure students' understanding of the dynamics of abusive relationships and ascertain whether this differs across program of study. Students in the schools of Nursing, Medicine and Rehabilitation attending a one-day workshop on IPV completed a brief survey in order to provide some preliminary data to address these study questions.

## Methods

A daylong interprofessional workshop on IPV is held annually at a large university in Ontario, Canada. Students from the Schools of Nursing, Medicine and Physical Rehabilitation attend the mandatory workshop. Students are warned of the potential for disturbing material and offered on-site resources. Counselors attend the workshop and are available to assist any student in immediate need of support. A voluntary brief, confidential Likert-scale survey was distributed to students at the morning break during the one-day workshop over the three study years (2003-2005). The medical and rehabilitation students were in their second year of graduate training, the nursing students were in the third year of an undergraduate program. Attendees answered basic demographic questions about age, country of origin and current school. They were also asked about any prior training pertaining to IPV. We included two questions about students' personal experience and history of witnessing of IPV:

1) "Have you ever been physically abused by an intimate partner?"

2) "Have you ever directly witnessed physical abuse in a relationship?"

We also queried students about their level of comfort with inquiry about IPV. Response categories included "strongly agree", "agree", "neutral", "disagree" and "strongly disagree". A dichotomous variable for comfort was created with the two agreement categories being used to model presence of comfort in addressing IPV. Summary and descriptive statistics were performed to examine basic demographic characteristics, attitudes toward and prevalence of IPV. The secondary aim of characterizing students' understanding of abusive relationship dynamics was addressed by measuring agreement with the statement, "I don't understand why victims remain in abusive relationships." Bivariate analyses examined whether rates of IPV varied by gender, country of origin and school. We examined potential predictors of student comfort with inquiry for IPV using logistic regression analysis. This model was adjusted for age, gender, country of birth; prior training for IPV, school, year the survey was taken and history of IPV or being a witness to IPV. The University Research Ethics Board approved the study. All analyses were conducted using SAS Version 9.1 (Cary, N.C.).

## Results

Over a three-year period, a total of 552 students completed the survey; 37% of the students were medical students, 33% were rehabilitation students and the remaining 30% were nursing students (Table 1). The overall response rate was 73%. The majority of the health professions students attending the workshops over the three years were female (n = 415/552, 76%). Most of the students reported no prior IPV training (n = 338, 61%); for those who had training, the most common source was undergraduate education (n = 86/214, 40%). Medical students had the highest rate of previous training. (Table 1)

**Table 1:** Characteristics of Students by Professional School N = 552

**Variable**	**Medical** **N = 208 (%)**	**Rehabilitation** **N = 181 (%)**	**Nursing** **N = 163 (%)**	**P value**
**Mean Age (SD)**^§^	24 (3.8)	25 (2.6)	21 (4.5)	< .0001
**Gender (REFERENCE = female)**	103 (50%)	158 (87%)	154 (94%)	< .0001
**Born in Canada**	175 (84%)	159 (88%)	137 (85%)	.51
**Prior IPV training** **(REFERENCE = none)**	139 (67%)	103 (57%)	96 (59%)	.11
**Personal IPV history**	18 (9%)	11 (6%)	9^‡ ^(5.5%)	.44
**Witnessed IPV**	50 (24%)	37* (21%)	36 (22%)	.71
**Summary of Attitudes and Comfort: percentage responding Agree Strongly or Agree**				
**"I don't understand why victims remain in abusive relationships."**	36 (17%)	38 (21%)	19 (12%)	.02
**"It is my role to intervene if a patient has been abused."**	171 (82%)	145 (80%)	136 (83%)	.24
**"I feel comfortable asking patients about IPV."**	99 (48%)	56 (31%)	78 (48%)	< .0001

^§^Standard deviation is given in parentheses for age variable only.

^‡ ^Missing 1 response on this question only (n = 162)

* Missing 1 response on this question only (n = 180)

The majority of students (82%) in all schools expressed the belief that it was their role to intervene on behalf of abused patients (Table 1), but the rehabilitation students expressed a lower self-report of comfort level than both nursing and medical students (Table 2). Similarly, the rehabilitation students were more likely to endorse a lack of understanding as to why someone would remain in an abusive relationship (Table 1). In the adjusted analyses, the only significant predictor of student-reported comfort was enrollment in either nursing or medical school. Gender, age, prior report of IPV training, year of workshop attendance and personal history of IPV were not predictors of comfort with IPV inquiry (Table 2).

**Table 2:** Predictors of Student Reported Comfort with IPV Inquiry

**Covariate**	**Unadjusted O.R. (95% C.I.)**	**Adjusted O.R. (95% C.I.)**
**Gender** **(REFERENCE = Female)**	1.08 (0.73-1.59)	1.34 (0.84-2.12)
**Age**	0.99 (0.95-1.03)	1.01 (0.96-1.06)
**Prior Training** **(REFERENCE = none)**	0.76 (0.54-1.08)	0.74 (0.52-1.06)
**Year of workshop attendance**	1.17 (0.95-1.43)	1.09 (0.88-1.35)
**School** **(REFERENCE = Rehab)**	0.49 (0.33-0.75)	0.45 (0.28-0.70)
**Country of Birth** **(REFERENCE = Canada)**	1.073 (0.66-1.73)	1.10 (0.67-1.81)
**Witness IPV**	0.68 (0.45-1.01)	0.76 (0.49-1.17)
**Lifetime IPV**	0.50 (0.26-0.98)	0.64 (0.31-1.33)

Overall, a total of 38 students (7%) reported lifetime IPV; the majority of these were female (n = 30/38, 79%). However, this was not statistically significant (χ^2 ^= 3.77, p = .15). Medical students had the highest rate of lifetime IPV (Table 1). Fewer foreign-born students (4%) reported IPV than did their Canadian counterparts (7%).

One fifth of all the students witnessed IPV at some point in their lives; the highest percentage was again found among medical students (Table 1). More female students reported witnessing IPV, however the difference was not statistically significant (χ^2 ^= 3.63, p = 0.16).

## Discussion

While the majority of students in our study agreed that it is their role to address IPV in clinical practice, knowledge and attitudes varied across schools. Age, prior training and even personal exposure to IPV did not change the relationship between field of study and comfort level with this issue. Rehabilitation students expressed lower comfort levels that may, in part, correspond to their report of less prior IPV training; however in logistic regression analysis, field of study remained a significant predictor of comfort even when prior training was controlled for. While rehabilitation students clearly viewed addressing IPV as part of their professional purview, their expressed comfort level and understanding of the dynamics of abusive relationships lagged behind those of nursing and medical students. This finding is unlikely due to level of study alone since rehabilitation students were second year postgraduate students comparable in age to the medical students, while the nursing students were younger undergraduates. Literature searches reveal a relative lack of publication in this field (compared to medicine and nursing) which may contribute to reduced awareness and familiarity among those entering this field. Personal exposure to IPV was a significant predictor of reduced comfort in unadjusted analyses, but this relationship did not remain significant with adjustment for potential confounders.

While prior work has shown that female medical students were more likely than their male counterparts to report prior IPV exposure, [18] this finding did not achieve statistical significance in our study. Interestingly, the school reporting the highest rates of IPV (Medicine) was also the group with the highest percentage of male students. Rates of exposure to lifetime IPV are known to increase with age, but in our study, age alone was unlikely responsible for medical students' higher reported IPV rates since the mean age of rehabilitation students was comparable.

It is notable that the rate of lifetime IPV measured in this study is significantly lower than that reported in Canadian population studies but comparable to estimates among U.S student cohorts. One possible explanation for this is the "healthy worker effect" theory, which posits that those with abuse histories may have lower educational attainment due to the adverse effects of the abuse, and thus be less likely to participate in professional training, lowering the rate of IPV in such populations. [28,30] Another potential contributing factor is underreporting of abuse history by students due to our administration of the survey in an open lecture hall with proximate seating of other students.

Foreign-born students' reported rates of IPV are similar to those of Canadian- born students. Since we did not query length of residency in Canada, we were unable to assess the level of acculturation of these students which may impact rates of IPV. Lower rates of IPV have been found among foreign born women in population-based studies in Canada [2] but the foreign born students enrolled in Canadian professional schools likely have higher levels of language proficiency and literacy than their counterparts in the general population.

### Limitations

This study has a number of limitations. Because the survey was administered during the workshop, students may have had privacy concerns when completing it, possibly resulting in response bias. We could not query 12 month (current) IPV separately because students attending the training who had intimate relationships with fellow students could have been seated together in the lecture hall, limiting the safety of inquiry about current IPV. Selection bias may have occurred because questionnaire completion was voluntary. Another concern is our measurement of IPV. Due to the need for brevity, we used one question to ascertain prior exposure to physical IPV and one to query witnessing IPV. Neither question has been validated. The lack of questions about emotional abuse also likely underestimated the true prevalence of IPV in this population. The students from the different schools were all at different levels in their training, thus unmeasured effects of clinical experience could have impacted some of their expressed knowledge and attitudes about IPV. Moreover, interprofessional educational initiatives remain unusual, so the findings from this study may not readily generalize to other more traditional teaching settings.

## Conclusion

Our study presents novel data regarding Canadian professional students and IPV which may aid educators developing curriculum in this field. While the majority of all students believed that it was their role to address IPV; further study of rehabilitation students, who will go on to work with vulnerable populations, is needed to explain why this group differs in expressed comfort and understanding of the dynamics of abusive relationships.

While we may have underestimated the true prevalence of IPV in this cohort, our study affirms that a proportion of Canadian health professions students are likely to have experienced IPV. IPV may also be a more salient issue for male students than previously described. Our findings require replication with validated, confidential measures. Study of this issue across Canadian institutions could also better inform educational initiatives in this challenging field. Future work should examine which teaching methods may be most effective for learners who have been victims or witnesses to IPV.

## Competing interests

The authors declare that they have no competing interests.

## Authors' contributions

MG assisted with the design of the survey, carried out the statistical analyses and drafted the manuscript. AT developed and ran the workshop, assisted with design of the survey, oversaw administration of the survey and helped to draft the manuscript. Both authors read and approved the final manuscript.
